# Prognostic Utility of a New Risk Stratification Protocol for Secondary Prevention in Patients Attending Cardiac Rehabilitation

**DOI:** 10.3390/jcm11071910

**Published:** 2022-03-30

**Authors:** Ignacio Cabrera-Aguilera, Consolació Ivern, Neus Badosa, Ester Marco, Xavier Duran, Diana Mojón, Miren Vicente, Marc Llagostera, Nuria Farré, Sonia Ruíz-Bustillo

**Affiliations:** 1 Unit of Biophysics and Bioengineering, Faculty of Medicine and Health Sciences, Universitat de Barcelona, 08036 Barcelona, Spain; ignaciocabrera.a@gmail.com; 2 Heart Diseases Biomedical Research Group, IMIM (Hospital del Mar Medical Research Institute), 08003 Barcelona, Spain; civern@psmar.cat (C.I.); nbadosa@psmar.cat (N.B.); sruiz@psmar.cat (S.R.-B.); 3 Department of Human Movement Sciences, School of Kinesiology, Faculty of Health Sciences, Universidad de Talca, Talca 3460000, Chile; 4 Cardiac Rehabilitation Unit, Department of Cardiology, Hospital del Mar, 08003 Barcelona, Spain; dmojon@psmar.cat (D.M.); 63592@parcdesalutmar.cat (M.V.); mllagostera@psmar.cat (M.L.); 5 Cardiac Rehabilitation Unit, Physical Medicine and Rehabilitation Department, Parc de Salut Mar (Hospital del Mar—Hospital de l’Esperança), 08003 Barcelona, Spain; emarco@psmar.cat; 6 Rehabilitation Research Group, Hospital del Mar Medical Research Institute, 08003 Barcelona, Spain; 7 Department of Medicine, Universitat Autònoma de Barcelona, 08003 Barcelona, Spain; 8 Methodological and Biostatistical Advisory Service, IMIM (Institut Hospital del Mar d’Investigacions Mèdiques), 08003 Barcelona, Spain; xduran@imim.es; 9 Department of Medicine, Universitat Pompeu Fabra, 08003 Barcelona, Spain

**Keywords:** acute coronary syndrome, ischemic heart disease, cardiac rehabilitation, exercise training, event-free survival, risk stratification

## Abstract

Several risk scores have been used to predict risk after an acute coronary syndrome (ACS), but none of these risk scores include functional class. The aim was to assess the predictive value of risk stratification (RS), including functional class, and how cardiac rehabilitation (CR) changed RS. Two hundred and thirty-eight patients with ACS from an ambispective observational registry were stratified as low (L) and no-low (NL) risk and classified according to exercise compliance; low risk and exercise (L-E), low risk and control (no exercise) (L-C), no-low risk and exercise (NL-E), and no-low risk and control (NL-C). The primary endpoint was cardiac rehospitalization. Multivariable analysis was performed to identify variables independently associated with the primary endpoint. The L group included 56.7% of patients. The primary endpoint was higher in the NL group (18.4% vs. 4.4%, *p* < 0.001). After adjustment for age, sex, diabetes, and exercise in multivariable analysis, HR (95% CI) was 3.83 (1.51–9.68) for cardiac rehospitalization. For RS and exercise, the prognosis varied: the L-E group had a cardiac rehospitalization rate of 2.5% compared to 26.1% in the NL-C group (*p* < 0.001). Completing exercise training was associated with reclassification to low-risk, associated with a better outcome. This easy-to-calculate risk score offers robust prognostic information. No-exercise groups were independently associated with the worst outcomes. Exercise-based CR program changed RS, improving classification and prognosis.

## 1. Introduction

Acute coronary syndrome (ACS), one of the main manifestations of ischemic heart disease (IHD), is a leading cause of death worldwide [1]. Many advances in pharmacological and non-pharmacological treatment (e.g., ST-elevation myocardial infarction primary angioplasty initiatives) have been achieved [2]. However, morbidity and mortality remain high. In addition, several risk scores have been used to predict risk in patients with ACS [3,4]. However, these risk scores are mainly based on in-hospital parameters such as Killip–Kimball class, ST-segment abnormalities, and cardiac biomarkers, among other clinical parameters. Remarkably, none of these risk scores include functional class. In addition, cardiorespiratory capacity estimated by metabolic equivalent (METS) has been consistently associated with prognosis; in ischemic heart disease, patients with poor cardiorespiratory capacity have a much worse prognosis [5,6]. Similarly, left ventricular ejection fraction (LVEF), a well-known prognosis factor, is not always included in the risk scores most used.

Cardiac rehabilitation (CR) after an ACS has a class I indication. However, CR remains widely underused. There are several reasons for this underuse. Patients identify distance, work responsibilities, lack of time, transportation problems, and comorbidities as the most significant barriers to enrolment [7]. Another limitation is logistics, as supervised exercise by physiotherapists, rehabilitation physicians, and cardiologists might not be available in all healthcare settings. To overcome this limitation, there have been several attempts to identify low-risk patients who could perform the unsupervised exercise at home or in a primary care setting. The Spanish Society of Cardiology (SSC) developed a CR protocol stratifying patients based on several parameters. The most relevant parameters were obtained from the echocardiogram and the exercise stress test during hospitalization or after hospital discharge [8]. Patients were categorized as low, mid, and high risk according to this classification. However, the SSC-EXCELENTE cardiac rehabilitation committee later proposed classifying patients only as low (L) or no-low risk (NL). This classification is consistent with that of other international entities and studies that indicate that all patients who are not at low risk should be considered high risk [9,10,11]. This stratification helps decide where the patient will perform the exercise training, but, surprisingly, it is unknown whether this simple classification can help identify patients with a bad prognosis.

Finally, completing the exercise training of a CR program is associated with an improvement in cardiorespiratory capacity measured by an increase in the METS achieved in the exercise stress test and has also been associated with an increase in LVEF [12,13]. Whether the improvements in function class and LVEF lead to a change in the low vs. no-low risk stratification (RS) and prognosis is substantially unknown.

Hence, the study aimed to assess whether an easy-to-calculate RS could identify patients with a worse prognosis, and how exercise-based CR changed this stratification.

## 2. Materials and Methods

### 2.1. Study Design, Population and Study Variables

After an ACS, all patients from the Hospital del Mar reference area are referred to the cardiac rehabilitation unit. From November 2016 to September 2019, 497 were assessed at the cardiac rehabilitation unit and included in the Ambispective Risk Optimization—Acute Coronary Syndrome (Risk-Op-ACS) registry (ClinicalTrials.gov Identifier: NCT03619395). We included patients with ST-elevation acute myocardial infarction (STEMI), non-ST-elevation acute myocardial infarction (non-STEMI), and unstable angina (UA). The diagnosis was made following the European Society of Cardiology guidelines [14,15]. The main exclusion criteria in the CR unit were patients from other health areas, those with a severe language barrier, or patients who refused to participate. For the present study, we only included patients with an assessment of cardiorespiratory capacity by exercise stress testing both at baseline and at the end of the rehabilitation program [16]. Thus, the final study sample included 238 patients.

We collected baseline demographic and clinical data and follow-up events. Follow-up was performed by directly contacting patients or relatives or reviewing medical records.

### 2.2. Cardiac Rehabilitation Program

The cardiac rehabilitation unit at the Hospital del Mar is an interdisciplinary program that combines interventions performed by cardiologists, nurses, rehabilitation physicians, physiotherapists, and psychiatrists. Detailed information on the unit’s characteristics has been previously described [16]. Briefly, all patients with an ACS receive education by specialized nurses on healthy habits during the ACS hospitalization, at 3 and 12 months after inclusion. Patients attend weekly group sessions with healthcare professionals to reinforce their health education. Finally, all patients are referred to participate in the exercise training component. Cardiorespiratory fitness is assessed at enrollment by a treadmill stress test. According to the patients’ characteristics, treadmill stress test and RS, rehabilitation physicians prescribe the setting, level, and type of exercise recommended to each patient. The ET intervention consists of 25 one-hour sessions, five times per week for five weeks. After five-minute warm-up and conditioning, each session begins with thirty minutes of exercise on a cycle ergometer at 80% of effort assessed by cardiorespiratory capacity test followed by twenty minutes of strength and resistance training of both upper and lower extremities with a load of 10 repetitions maximum (10 RM) and ending with a period of five minutes cool-down. The workload progression is adjusted weekly according to the patient’s tolerance by the Borg perceived effort scale. Sessions are supervised by an expert physiotherapist, using continuous heart rate and pulse oximetry monitoring. Once the patients complete the 25 sessions, a treadmill stress test is carried out to re-evaluate cardiorespiratory functional status. In patients with reduced baseline LVEF, an echocardiogram is repeated during follow-up at the discretion of their treating cardiologist.

### 2.3. Cardiac Risk Stratification Process

All patients were stratified according to the risk level score developed by the Spanish Society of Cardiology (SSC) and the recommendation of the SSC-EXCELENTE committee of cardiac rehabilitation [8,9]. Patients in the no-low group had one or more of these parameters: cardiorespiratory capacity <7 METs, angina during the stress test, ST depression >2 mm with heart rate <135 bpm, hypotensive response to exercise or malignant ventricular arrhythmias, reversible wall defects with stress thallium, reinfarction, residual ischemia, depression/anxiety, frailty, history of decompensated heart failure during ACS admission, and LVEF <49%. Patients in the low group comprised all the other patients. The main goal of this classification is to identify low-risk patients who can safely complete the ET component of the cardiac rehabilitation in a setting other than the hospital and, thus, make ET more accessible to all the patients who may benefit from it.

Some patients did not complete the exercise training program for several reasons and were considered the control group. Therefore, we classified the patients into four groups according to exercise compliance and risk stratification: low risk and exercise (L-E), low risk and no exercise (control) (L-C), no-low risk and exercise (NL-E), and no-low risk and no exercise (control) (NL-C).

### 2.4. Aims and Endpoint

The study’s primary aim was to evaluate whether a low and no-low risk stratification can help predict outcomes in patients with a recent ACS. The primary endpoint was cardiac rehospitalization. Cardiac rehospitalization was defined as any cardiac event that required hospital admission for more than 24 h and included: arrhythmias, heart failure, SCA, and unplanned coronary revascularization.

The secondary endpoints were to evaluate whether this risk stratification is modified by participating in exercise training (ET) and to assess whether changes in the group risk classification over time affect prognosis. Finally, we evaluated whether a low and no-low risk stratification was associated with the primary endpoint’s individual endpoints.

### 2.5. Ethics

The study was designed in compliance with the ethical principles set forth by the Declaration of Helsinki. The data included in this study incorporated both data from a prospective and retrospective registry. The prospective registry was carried out from July 2018 to September 2019. The Ethics Committee of the Hospital del Mar (Parc de Salut Mar) approved the study (N° 2018/7896/I), and all patients provided written informed consent. To increase the sample size, and given that the same protocol had been carried out before, the Ethics Committee approved including patients from November 2016 to June 2018 retrospectively. It waived the need for written informed consent in this group.

### 2.6. Statistics

Data for continuous variables were expressed as mean ± standard deviation (SD) or median and interquartile range (IQR) based on normality distribution assessed by Kolmogorov–Smirnov test or Shapiro–Wilk test for smaller groups. Categorical variables were expressed as numbers (n) and percentages (%). Differences in baseline characteristics and risk variation between groups previously defined by risk stratification were tested using Chi-square, Student’s *t*, or Mann–Whitney U test as needed. For baseline characteristics between groups previously defined by exercise compliance and risk stratification, one-way analysis of variance or Kruskal–Wallis tests were used. Minimal detectable change was calculated following previous studies [17]. Univariable and multivariable analyses were performed using the Cox proportional hazard regression model to examine the association between risk groups and cardiac rehospitalization. Variables with an overall significance value of *p* < 0.05 were entered for multiple Cox regression analysis to identify the strongest predictors for event-free survival. We also included sex, as it is a well-known risk factor. The model was adjusted for potential confounders selected by stepwise forward inclusion, among patient characteristics previously defined.

The number of events per degree of freedom was fairly small, and below the rule of thumb established at 10 events per variable. However, the convenience of this rule of thumb has been largely discussed in the literature in recent years [18]. The proportional hazard assumption, checked by examining residuals (for overall model and variable by variable), was not violated. The log-rank test was performed to compare Kaplan–Meier survival curves. Differences from baseline to follow-up in RS were evaluated using the McNemar test. All analysis was performed using IBM SPSS Statistics v25 (Armonk, NY, USA) and GraphPad Prism 8.0 (San Diego, CA, USA). For all tests, *p* < 0.05 was considered as statistically significant.

## 3. Results

Baseline clinical characteristics of the study population divided by risk groups are summarized in Table 1. In brief, most participants were middle-aged men admitted due to STEMI and who had a one-vessel disease and preserved ejection fraction. Overall, 56.7% of the patients were in the low-risk group. The main differences between groups were age (59.3 years in the low risk vs. 63.3 years in the no-low risk, *p* = 0.006), ejection fraction (51 vs. 60%, respectively, *p* < 0.001), diabetes mellitus (15.6 vs. 27.2%, *p* = 0.028) and glycated hemoglobin levels (5.6 vs. 5.7%, *p* = 0.007). Supplementary Table S1 shows the difference between patients included in this study and patients who were not.

The median (interquartile range) absolute METs gained in the whole cohort who completed the ET was 1.0 (0.2–2.0). As summarized in Table 2, METs achieved were higher in the low-risk group at baseline and follow-up. Patients in the no-low risk group significantly increased the METs gained (Table 2).

**Figure 1:**
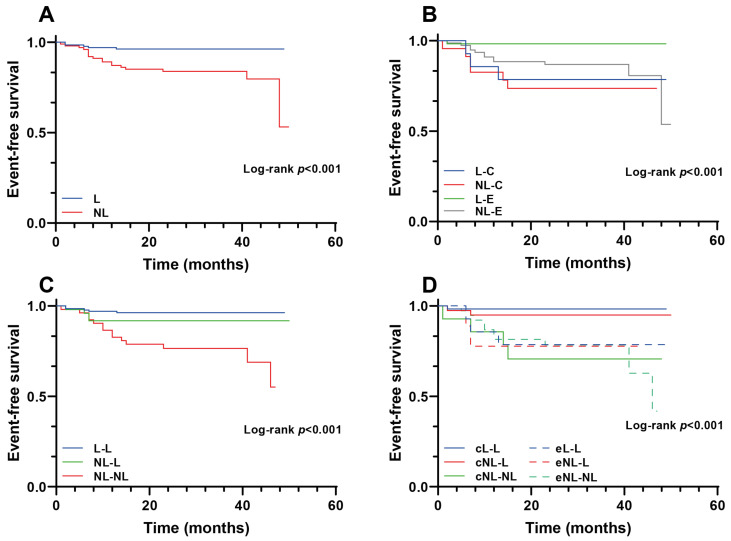
Kaplan–Meier curves according to the different risk groups. Panel (**A**): Global risk stratification. Panel (**B**): Exercise compliance classification. Panel (**C**): Rehabilitation risk stratification variation. Panel (**D**): Rehabilitation exercise compliance risk stratification variation. L, Low-risk group; NL, No-low risk group; C, Control; E, exercise; L-L, low to low risk; NL-L, No-low to low risk; NL-NL, No-low to no-low risk.

The median follow-up was 31 (23–39) months. Table 2 and Table 3 show that the primary endpoint of cardiac rehospitalization was higher in the no-low risk group (18.4% vs. 4.4%, *p* < 0.001, univariable hazard ratio (HR) (95% confidence interval (CI): 4.32 (1.73–10.82)). In multivariable analysis, after adjustment for age, sex, diabetes mellitus, and the completion of the exercise training, the HR (95% CI) was 3.83 (1.51–9.68) (Table 3). Figure 1A shows the Kaplan–Meier survival curve with a better prognosis in the low-risk group. Supplementary Table S2 shows the difference between patients with cardiac rehospitalization and patients without. Interestingly, the only differences were the presence of hyperlipidemia, anemia, and the number of coronary arteries affected.

Table 4 shows the differences in baseline characteristics and prognosis according to the risk group and the completion of the exercise training. Only 15.6% were in the control group, and of those, 37.8% were in the low-risk group, and 62.2% were in the no-low group. In the exercise group, 60% were in the low-risk group and 39.8% in the no-low risk group. Interestingly, the only differences between groups were age and glycated hemoglobin. Outcomes were different (Table 5 and Figure 1B), with cardiac rehospitalization of 2.5% in the low-risk exercise group compared to 26.1% in the no-low no-exercise (control) group (*p* < 0.001).

**Figure 2:**
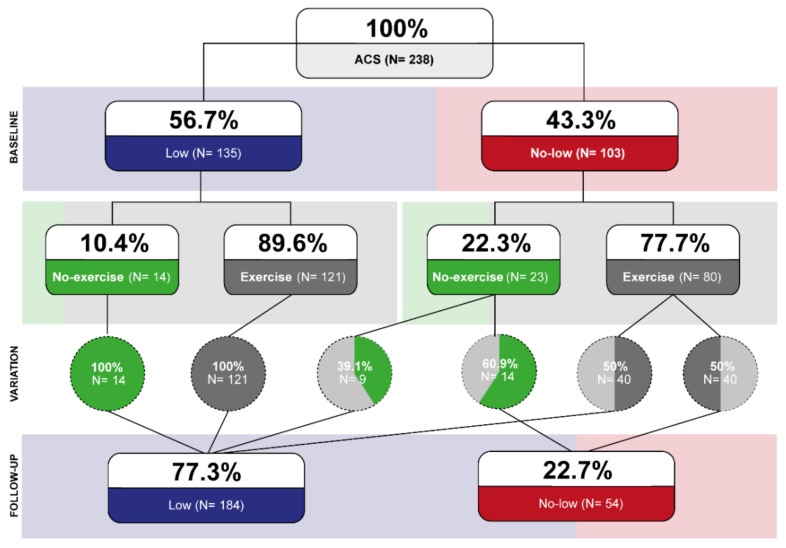
Risk stratification variation.

Figure 2 shows the change in the risk classification after the training exercise (or repeated treadmill exercise stress test in the control group). Significant increase (from 56.7% to 77.3%, *p* < 0.001) and decrease (from 43.3% to 22.7%, *p* < 0.001) were observed in low and no-low groups respectively. Participation in the exercise training was associated with a significantly higher proportion of patients classified as low risk (McNemar test, *p* < 0.001). All patients in the low-risk group (both in the control and exercise group) remained at low risk. Of patients in the no-low risk group, 60.9% remained in the no-low risk group in the control group, compared with 50% of patients who remained in the no-low risk group in the exercise group. Figure 1C shows that the patients who remained in the no-low group had a worse prognosis. However, patients whose functional class improved enough to be in the low-risk group had a similar outcome to those who were always in the low-risk group. Figure 1D shows that patients in the exercise group who changed to low risk had an excellent prognosis. Those who remained in the no-low risk group had a prognosis similar to the control group.

## 4. Discussion

One of the most relevant findings from this study was that 56.7% of patients who had been admitted due to an ACS were low-risk according to an easy-to-calculate score that included cardiorespiratory capacity and left ventricular ejection fraction. Cardiac rehospitalization in the no-low risk group was significantly higher than in the low-risk group (HR 3.83 (95 CI 1.51–9.68)). The completion of the exercise program was also independently associated with a better prognosis, and 50% of the patients in the no-low risk group who completed it became low-risk.

Baseline characteristics did not differentiate patients in the low- and no-low risk groups. The only differences were age and diabetes, but these variables were not strikingly different. Indeed, the mean age was 61.0 ± 10.5, and there was only a 4-year difference between groups. In some of the most used risk scores, this difference would not have made significant changes in stratification. Indeed, the GRACE and TIMI score considers age a risk when the patient is older than 65 years [19,20,21]. Diabetes is a significant risk factor among patients suffering from a myocardial infarction. It is included in the GRACE score [22], and glycated hemoglobin after an ACS might also predict future events [23,24]. In our study, diabetes was more prevalent in the no-low risk group (27.2 vs. 45.6%, *p* = 0.028). Although glycated hemoglobin was statistically higher in the no-low risk group, the differences were clinically not relevant (5.7% (5.4–6.7) vs. 5.6% (5.3–5.8), *p* = 0.007). Compared to patients who did not decide to participate in the ET, patients in the present study were younger, less frequently female, and with less past medical history of hypertension, diabetes mellitus, chronic kidney disease, anemia, and past acute coronary syndrome or acute coronary syndrome myocardial infarction. These differences are consistent with previous literature [25].

As expected, due to the variables included in the stratification protocol, LVEF and cardiorespiratory capacity differed between both groups. However, it is worth noting that the median LVEF was >50% in both groups. Interestingly, although patients in the low-risk group had higher METs (10.3 vs. 8.3, *p* < 0.001), both groups had good cardiorespiratory capacity. Several studies have shown that METs vary with age, but patients in their 50 s and 60 s have a cardiorespiratory capacity of 6 to 10 METs [26,27]. Our cohort comprised middle-aged males with a high prevalence of risk factors but a relatively low prevalence of comorbidities and previous ACS. Moreover, most patients had one-vessel disease and presented with STEMI. Therefore, it is likely that this cohort was reasonable fit before the ACS. After completing the ET, there was an increase in the relative increase and total METs achieved in the treadmill stress test. Although not significant, there was also an increase in the absolute increase of METs achieved by the four groups (0.3 to 1.3 METS in the low-risk control and no-low risk and ET). This absolute increase is similar to the 0.52 to 1.55 METs increase described in other studies and meta-analyses [28]. It also compares favorably to the 0.21 minimal detectable change expected.

The low-risk patients had a cardiac rehospitalization rate much lower than the no-low risk group (4.4% vs. 18.4%, *p* < 0.001). After multivariable analysis, patients in the no-low risk group had a worse prognosis with an HR 3.83 (95% CI 1.51–9.68) for the primary endpoint. Other risk scores have shown that prognosis after an ACS worsens with increased risk. Risk stratification of the GRACE score indicated that the mortality risk of the intermediate-risk and high-risk groups was higher with an HR 3.23 (1.59–6.55) for the intermediate-risk group and HR 15.31 (4.43–51.62) for the highest risk group. Similar results were observed with MACCE risk [29]. Still, different endpoints, follow-up periods, and baseline characteristics can explain the differences in outcomes with our results, especially in the high-risk group. Hospitalization due to heart failure was infrequent in our cohort (two patients, 1.7%) and was numerically much higher in the no-low risk group. Five percent of the patients experienced hospitalization due to an arrhythmic event, which was more frequent in the no-low risk group. This finding is consistent with previous reports that showed benefits of CR in patients with arrhythmia [30].

Finally, this study showed that all patients initially classified in the low-risk group remained in this group. However, completion of the exercise training was associated with reclassification from the no-low to low-risk group more frequently than in the control group. The no-low risk group who completed the exercise training had the most significant improvement with a relative increase of 14.3% in the METS achieved in the treadmill stress test. This reclassification was associated with a better outcome. Although several studies and meta-analyses have shown that exercise training is associated with a better prognosis [31,32,33], few studies have analyzed whether the change in risk categories is associated with prognosis. The proposed risk score could identify no-low risk patients soon after an ACS and add further prognostic information after an exercise training program.

The main limitation of this study is that the results might not apply to other settings as a single-center observational study. Some of the patients were included retrospectively, which might lead to bias. However, we believe that the risk of bias is low since all patients followed the same protocol and that the information was documented in the medical record in a structured way

## 5. Conclusions

Given that this easy-to-calculate routine risk stratification method offers prognostic information, it should be used in all patients after an ACS. Low-risk patients had an excellent prognosis compared to the no-low risk group. Exercise-based CR program showed the ability to change risk stratification, improving functional classification and prognosis of these patients who initially belonged to the no-low risk and ended as low risk. Therefore, this risk stratification score could identify patients suitable for exercise training in an unsupervised setting and identify low-risk patients with excellent prognosis at follow-up.

## Figures and Tables

**Table 1:** Baseline characteristics according to the low and no-low groups.

Characteristic/Variable	L (*n* = 135)	NL (*n* = 103)	*p*-Value
Anthropometric			
Age (years)	59.3 ± 10.2	63.3 ± 10.6	0.006
Women	19 (14.1)	17 (16.5)	0.604
BMI (kg/m^2^)	27.3 (24.7–29.3)	26.9 (24.9–30.5)	0.631
Risk factors and comorbidities			
Hypertension	64 (47.4)	56 (54.4)	0.287
Hyperlipidemia	92 (68.1)	65 (63.1)	0.416
Diabetes mellitus	21 (15.6)	28 (27.2)	0.028
Current smoker	59 (43.7)	33 (32.0)	0.288
Previous smoker > 1 year	42 (31.1)	35 (34.0)	
Previous smoker < 1 year	5 (3.7)	5 (4.9)	
COPD	5 (3.7)	10 (9.7)	0.059
Cerebrovascular disease	3 (2.2)	7 (6.8)	0.081
Peripheral vascular disease	6 (4.4)	3 (2.9)	0.539
Anemia	13 (9.3)	13 (12.6)	0.464
Chronic kidney disease	3 (2.2)	5 (4.9)	0.264
Diagnostics			
STEMI	60 (40.4)	46 (44.7)	0.684
NSTEMI	54 (40.0)	37 (35.9)	
Unstable angina	21 (15.6)	20 (19.4)	
Previous ACS-MI	17 (12.6)	18 (17.5)	0.292
One vessel disease	84 (62.2)	51 (49.5)	0.133
Two vessels disease	29 (21.5)	26 (25.2)	
Three vessels disease	17 (16.2)	24 (23.3)	
Ejection fraction (%)	60 (55.0–63.5)	51 (43.5–60.0)	<0.001
Exercise testing			
METs	10.3 (9.1–12.4)	8.3 (6.6–9.8)	<0.001
Maximum predicted heart rate (%)	81 (73.0–89.0)	79 (67.0–87.0)	0.073
Peak systolic blood pressure (mmHg)	154 (142–173)	148 (132–165)	0.055
Blood test			
Glucose, mg/dL	104 (94–124)	116 (99–151)	0.005
Glycated hemoglobin, %	5.6 (5.3–5.8)	5.7 (5.4–6.7)	0.007
LDLc, mg/dL	117 (90–136)	107(81–136)	0.323

Data are mean ± SD, median (IQR), or numbers (n) and percentages (%). L, Low group; NL, No-low group; ACS, acute coronary syndrome-myocardial infarction; BMI, body mass index; COPD, chronic obstructive pulmonary disease; CV, cardiovascular; METs, metabolic equivalent; MI, myocardial infarction; NSTEMI, non-ST-elevation myocardial infarction; STEMI, ST-elevation myocardial infarction.

**Table 2:** Outcomes according to the low and no-low groups.

Outcomes	L (*n* = 135)	NL (*n* = 103)	*p*-Value
Follow-up METs	11.8 (9.8–13.3)	9.8 (7.9–11.4)	<0.001
Relative increase in METs (%)	7.6 (0.0–17.4)	12.3 (2.6–27.4)	0.019
Absolute increase in METs	0.8 (0.0–1.9)	1.1 (0.2–2.1)	0.173
All causes re-admission	27 (20.0)	39 (37.9)	0.002
All causes death	0 (0.0)	2 (1.9)	0.104
Cardiac rehospitalization	6 (4.4)	19 (18.4)	<0.001
Heart failure	1 (0.7)	3 (2.9)	0.197
Arrythmias	2 (1.5)	10 (9.7)	0.004
Revascularization	4 (3.0)	9 (8.7)	0.052
New ACS	0 (0.0)	7 (6.8)	0.002

Data are median (IQR) or numbers (n) and percentages (%). L, Low group; NL, No-low group; ACS, acute coronary syndrome-myocardial infarction; METs, metabolic equivalent.

**Table 3:** Univariable and multivariable Cox regression analyses for cardiac rehospitalization.

	Univariable HR (95% CI)	*p*-Value	Adjusted HR (95% CI)	*p*-Value
No-low risk group	4.32 (1.73–10.82)	0.002	3.83 (1.51–9.68)	0.005
No-exercise group	3.15 (1.39–7.15)	0.006	2.52 (1.10–5.78)	0.021
Age (years)	1.03 (0.99–1.07)	0.103	-	
Men	1.22 (0.36–4.10)	0.745	-	
Mellitus diabetes	1.40 (0.47–4.02)	0.556	-	

**Table 4:** Baseline characteristics according to risk stratification and exercise training completion.

Characteristic/Variable	L-C (*n* = 14)	NL-C (*n* = 23)	L-E (*n* = 121)	NL-E (*n* = 80)	*p*-Value
Anthropometric					
Age (years)	61.4 ± 9.3	65.5 ± 12.1	59.8 ± 9.7	62.1 ± 10.1	0.005
Women	3 (21.4)	5 (21.7)	16 (13.2)	12 (15.0)	0.669
BMI (kg/m^2^)	26.7 (24.0–27.7)	28.4 (23.5–31.2)	27.3 (24.7–29.3)	26.7 (24.8–30.5)	0.830
Risk factors and comorbidities					
Hypertension	6 (42.9)	16 (69.6)	58 (47.9)	40 (50.0)	0.262
Hyperlipidemia	8 (57.1)	13 (56.5)	84(69.4)	52 (65.0)	0.557
Diabetes mellitus	1 (7.1)	8 (34.8)	20 (16.5)	20 (25.0)	0.088
Current smoker	9 (64.3)	7 (30.4)	50 (41.3)	26 (32.5)	0.478
Previous smoker > 1 year	3 (21.4)	6 (26.1)	39 (32.2)	29 (36.3)	
Previous smoker < 1 year	0 (0.0)	1 (4.3)	5 (4.1)	4 (5.0)	
COPD	1 (7.1)	2 (8.7)	4 (3.3)	8 (10.0)	0.269
Cerebrovascular disease	0 (0.0)	2 (8.7)	3 (2.5)	5 (6.3)	0.322
Peripheral vascular disease	1 (7.1)	0 (0.0)	5 (4.1)	3 (3.8)	0.710
Anemia	3 (21.4)	4 (17.4)	10 (8.3)	9 (11.3)	0.325
Chronic kidney disease	0 (0.0)	2 (8.7)	3 (2.5)	3 (3.8)	0.419
CV family history	3 (21.4)	4 (17.4)	26 (21.5)	12 (15.0)	0.702
Sudden death family history	0 (0.0)	1 (4.3)	6 (5.0)	1 (1.3)	0.457
Diagnostics					
STEMI	4 (28.6)	9 (39.1)	56 (46.3)	37 (46.3)	0.765
NSTEMI	7 (50.0)	8 (34.8)	47 (38.8)	29 (36.3)	
Unstable Angina	3 (21.4)	6 (26.1)	18 (14.9)	14 (17.5)	
Previous ACS-MI	3 (21.4)	7 (30.4)	14 (11.6)	11 (13.8)	0.109
One vessel disease	8 (57.1)	7 (30.4)	76 (62.8)	44 (55.0)	0.228
Two vessels disease	4 (28.6)	7 (30.4)	25 (20.7)	19 (23.8)	
Three vessels disease	2 (14.3)	9 (39.1)	15 (12.4)	15 (18.8)	
Ejection fraction (%)	62 (55.5–63.5)	58 (51.0–60.0)	60 (56.0–64.0)	51 (41.0–60.0)	<0.001
Exercise testing					
METs	9.8 (8.5–11.8)	6.8 (6.2–7.8)	10.3 (9.3–12.6)	8.5 (6.7–10.0)	<0.001
Maximum predicted heart rate (%)	80 (72.0–83.5)	82 (71.0–87.0)	81 (74.0–89.0)	76 (65.5–85.5)	0.088
Peak systolic blood pressure (mmHg)	155 (139–180)	160 (133–183)	154 (142–172)	147 (132–161.5)	0.103
Blood test					
Glucose, mg/dL	101 (87–112)	126 (106–190)	105 (95–124)	116 (97–151)	0.032
Glycated hemoglobin, %	5.6 (5.3–5.7)	6.7 (5.7–8.3)	5.6 (5.4–5.9)	5.6 (5.4–6.5)	0.016
LDL, mg/dL	115 (89–129)	102 (69–110)	118 (93–140)	111 (84–139)	0.127
Outcomes					
Follow-up METs	10.8 (8.3–12.2)	8.1 (6.0–10.8)	12.0 (9.8–13.3)	9.8 (8.3–11.5)	<0.001
Relative increase in METs (%)	4.5 (−4.3–16.7)	6.5 (−3.2–27.9)	7.7 (0.8–17.6)	14.3 (3.2–26.8)	0.039
Absolute increase in METs	0.3 (−0.4–1.0)	0.5 (−0.2–1.2)	0.9 (0.0–1.9)	1.3 (0.3–2.3)	0.119
Cardiac rehospitalization	3 (21.4)	6 (26.1)	3 (2.5)	13 (16.3)	<0.001
All causes readmission	7 (50.0)	12 (52.2)	20 (16.5)	27 (33.8)	<0.001
All causes death	0 (0.0)	1 (4.3)	0 (0.0)	1 (1.3)	0.195
Revascularization	3 (21.4)	3 (13.0)	1 (0.8)	6 (7.5)	0.002
New ACS	0 (0.0)	2 (8.7)	0 (0.0)	5 (6.3)	0.020

Data are mean ± SD, median (IQR), or numbers (n) and percentages (%). L-C, Low risk and control group; NL-C, No-low risk and control group; L-E, Low risk and exercise group; NL-E, No-low risk and exercise group; ACS, acute coronary syndrome-myocardial infarction; BMI, body mass index; COPD, chronic obstructive pulmonary disease; CV, cardiovascular; METs, metabolic equivalent; MI, myocardial infarction; NSTEMI, non-ST-elevation myocardial infarction; STEMI, ST-elevation myocardial infarction.

**Table 5:** Outcomes according to risk stratification and exercise training completion.

Outcomes	L-C (*n* = 14)	NL-C (*n* = 23)	L-E (*n* = 121)	NL-E (*n* = 80)	*p*-Value
Follow-up METs	10.8 (8.3–12.2)	8.1 (6.0–10.8)	12.0 (9.8–13.3)	9.8 (8.3–11.5)	<0.001
Relative increase in METs (%)	4.5 (−4.3–16.7)	6.5 (−3.2–27.9)	7.7 (0.8–17.6)	14.3 (3.2–26.8)	0.039
Absolute increase in METs	0.3 (−0.4–1.0)	0.5 (−0.2–1.2)	0.9 (0.0–1.9)	1.3 (0.3–2.3)	0.119
All causes readmission	7 (50.0)	12 (52.2)	20 (16.5)	27 (33.8)	<0.001
All causes death	0 (0.0)	1 (4.3)	0 (0.0)	1 (1.3)	0.195
Cardiac rehospitalization	3 (21.4)	6 (26.1)	3 (2.5)	13 (16.3)	<0.001
Heart failure	1 (7.1)	1 (4.3)	0 (0.0)	2 (2.5)	0.116
Arrythmias	0 (0.0)	3 (13.0)	2 (1.7)	7 (8.8)	0.029
Revascularization	3 (21.4)	3 (13.0)	1 (0.8)	6 (7.5)	0.002
New ACS	0 (0.0)	2 (8.7)	0 (0.0)	5 (6.3)	0.020

Data are median (IQR) or numbers (n) and percentages (%). L-C, Low risk and control group; NL-C, No-low risk and control group; L-E, Low risk and exercise group; NL-E, No-low risk and exercise group; ACS, Acute coronary syndrome-myocardial infarction; METs, metabolic equivalent.

## Data Availability

The data supporting the findings of this study are available from the corresponding author upon request.

## Supplementary Materials

The following supporting information can be downloaded at: https://www.mdpi.com/article/10.3390/jcm11071910/s1, Table S1: Baseline characteristics between patients who attended cardiac rehabilitation and the whole cohort of ambispective registry; Table S2: Baseline characteristics between patients who had cardiac readmission and those who did not.

## References

[1] Ab Khan M., Hashim M.J., Mustafa H., Baniyas M.Y., Al Suwaidi S.K.B.M., AlKatheeri R., Alblooshi F.M.K., Almatrooshi M.E.A.H., Alzaabi M.E.H., Al Darmaki R.S. (2020). Global Epidemiology of Ischemic Heart Disease: Results from the Global Burden of Disease Study. Cureus.

[2] Collet J.P., Thiele H., Barbato E., Barthélémy O., Bauersachs J., Bhatt D.L., Karia N. (2020). 2020 ESC Guidelines for the management of acute coronary syndromes in patients presenting without persistent ST-segment elevation. Eur. Heart J..

[3] D’Ascenzo F., Biondi-Zoccai G., Moretti C., Bollati M., Omedè P., Sciuto F., Presutti D.G., Modena M.G., Gasparini M., Reed M. (2012). TIMI, GRACE and alternative risk scores in Acute Coronary Syndromes: A meta-analysis of 40 derivation studies on 216,552 patients and of 42 validation studies on 31,625 patients. Contemp. Clin. Trials.

[4] Timóteo A.T., Rosa S.A., Nogueira M.A., Belo A., Ferreira R.C. (2016). Validação externa do score de risco ProACS para estratificação de risco de doentes com síndrome coronária aguda. Rev. Port. Cardiol..

[5] Tang W.H.W., Topol E.J., Fan Y., Wu Y., Cho L., Stevenson C., Ellis S.G., Hazen S.L. (2014). Prognostic value of estimated functional capacity incremental to cardiac biomarkers in stable cardiac patients. J. Am. Heart Assoc..

[6] McNeer J.F., Margolis J.R., Lee K.L., Kisslo A.J., Peter R.H., Kong Y., Behar V.S., Wallace A.G., McCants C.B., Rosati A.R. (1978). The role of the exercise test in the evaluation of patients for ischemic heart disease. Circulation.

[7] Winnige P., Filakova K., Hnatiak J., Dosbaba F., Bocek O., Pepera G., Papathanasiou J., Batalik L., Grace S.L. (2021). Validity and Reliability of the Cardiac Rehabilitation Barriers Scale in the Czech Republic (CRBS-CZE): Determination of Key Barriers in East-Central Europe. Int. J. Environ. Res. Public Health.

[8] Velasco J.A., Cosín J., Maroto J.M., Muñiz J., Casasnovas J.A., Plaza I., Abadal L.T. (2000). Guías de práctica clínica de la Sociedad Española de Cardiología en prevención cardiovascular y rehabilitación cardíaca. Rev. Española Cardiol..

[9] Procedimiento Rehabilitación Cardiaca—Sociedad Española de Cardiología. https://secardiologia.es/institucional/reuniones-institucionales/sec-calidad/sec-excelente/procedimientos/8722-procedimiento-rehabilitacion-cardiaca.

[10] American Association of Cardiovascular & Pulmonary Rehabilitation (2016). Guidelines for Cardiac Rehabilitation Programs.

[11] Da Silva A.K.F., da Costa de Rezende Barbosa M.P., Bernardo A.F.B., Vanderlei F.M., Pacagnelli F.L., Vanderlei L.C.M. (2014). Cardiac risk stratification in cardiac rehabilitation programs: A review of protocols. Rev. Bras. Cir. Cardiovasc..

[12] López-Aguilera J., Casado-Adam P., Heredia-Torres M.A., Mazuelos-Bellido F. (2015). Effectiveness of Cardiac Rehabilitation in Increased Left Ventricle Ejection Fraction and Cardiovascular Secondary Prevention. Int. J. Clin. Cardiol..

[13] Wang Y., Chien C.W., Xu Y., Tung T.H. (2021). Effect of Exercise-Based Cardiac Rehabilitation on Left Ventricular Function in Asian Patients with Acute Myocardial Infarction after Percutaneous Coronary Intervention: A Meta-Analysis of Randomized Controlled Trials. Healthcare.

[14] Ibanez B., James S., Agewall S., Antunes M.J., Bucciarelli-Ducci C., Bueno H., Caforio A.L.P., Crea F., Goudevenos A.J., Halvorsen S. (2018). 2017 ESC Guidelines for the management of acute myocardial infarction in patients presenting with ST-segment elevation. Eur. Heart J..

[15] Wang Y., Yan B.P., Nichol M.B., Tomlinson B., Lee V.W. (2016). 2015 ESC Guidelines for the management of acute coronary syndromes in patients presenting without persistent ST-segment elevationTask Force for the Management of Acute Coronary Syndromes in Patients Presenting without Persistent ST-Segment Elevation of the European Society of Cardiology (ESC). Eur. Heart J..

[16] Cabrera-Aguilera I., Ivern C., Badosa N., Marco E., Salas-Medina L., Mojón D., Vicente M., Llagostera M., Farré N., Ruiz-Bustillo S. (2021). Impact of and Reasons for Not Performing Exercise Training After an Acute Coronary Syndrome in the Setting of an Interdisciplinary Cardiac Rehabilitation Program: Results From a Risk-Op- Acute Coronary Syndrome Ambispective Registry. Front. Physiol..

[17] Bellet R.N., Francis R.L., Jacob J.S., Healy K.M., Bartlett H.J., Adams L., Morris N.R. (2016). Fast-track equivalent to traditional cardiac rehabilitation? Pilot study outcome. Eur. J. Physioterapy.

[18] Vittinghoff E., McCulloch C.E. (2007). Relaxing the rule of ten events per variable in logistic and Cox regression. Am. J. Epidemiol..

[19] Avezum A., Makdisse M., Spencer F., Gore J.M., Fox K.A., Montalescot G., Grace Investigators (2005). Impact of age on management and outcome of acute coronary syndrome: Observations from the global registry of acute coronary events (GRACE). Am. Heart J..

[20] Antman E.M., Cohen M., Bernink P.J.L.M. (2000). The TIMI risk score for unstable angina/non-ST elevation MI: A method for prognostication and therapeutic decision making. JAMA.

[21] Morrow D.A., Antman E.M., Charlesworth A., Cairns R., Murphy S.A., de Lemos J.A., Giugliano R.P., McCabe C.H., Braunwald E. (2000). TIMI Risk Score for ST-Elevation Myocardial Infarction: A Convenient, Bedside, Clinical Score for Risk Assessment at Presentation. Circulation.

[22] Fox A.A.K., Dabbous O.H., Goldberg R.J., Pieper K.S., Eagle A.K., Van de Werf F., Avezum A., Goodman S.G., Flather M.D., Anderson F.A. (2006). Prediction of risk of death and myocardial infarction in the six months after presentation with acute coronary syndrome: Prospective multinational observational study (GRACE). BMJ.

[23] Timmer J.R., Hoekstra M., Nijsten M.W., van der Horst I.C., Ottervanger J.P., Slingerland R.J., Dambrink J.-H.E., Bilo H.J., Zijlstra F., Hof A.W.V. (2011). Prognostic value of admission glycosylated hemoglobin and glucose in nondiabetic patients with ST-segment-elevation myocardial infarction treated with percutaneous coronary intervention. Circulation.

[24] Waters D.D., Arsenault B.J. (2016). Predicting Prognosis in Acute Coronary Syndromes: Makeover Time for TIMI and GRACE?. Can. J. Cardiol..

[25] Pardaens S., Willems A.-M., Clays E., Baert A., Vanderheyden M., Verstreken S., Du Bois I., Vervloet D., De Sutter J. (2017). The impact of drop-out in cardiac rehabilitation on outcome among coronary artery disease patients. Eur. J. Prev. Cardiol..

[26] Kokkinos P., Faselis C., Myers J., Sui X., Zhang J., Blair S.N. (2014). Age-specific exercise capacity threshold for mortality risk assessment in male veterans. Circulation.

[27] Morris C.K., Myers J., Froelicher V.F., Kawaguchi T., Ueshima K., Hideg A. (1993). Nomogram based on metabolic equivalents and age for assessing aerobic exercise capacity in men. J. Am. Coll. Cardiol..

[28] Sandercock G.R.H., Cardoso F., Almodhy M., Pepera G. (2013). Cardiorespiratory fitness changes in patients receiving comprehensive outpatient cardiac rehabilitation in the UK: A multicentre study. Heart.

[29] Zhao X., Li J., Xian Y., Chen J., Gao Z., Qiao S., Yang Y., Gao R., Xu B., Yuan J. (2020). Prognostic value of the GRACE discharge score for predicting the mortality of patients with stable coronary artery disease who underwent percutaneous coronary intervention. Catheter. Cardiovasc. Interv..

[30] Smart N.A., King N., Lambert J.D., Pearson M.J., Campbell J., Risom S.S., Taylor R.S. (2018). Exercise-based cardiac rehabilitation improves exercise capacity and health-related quality of life in people with atrial fibrillation: A systematic review and meta-analysis of randomised and non-randomised trials. Open Heart.

[31] Budts W., Pieles G.E., Roos-Hesselink J.W., Sanz de la Garza M., D’Ascenzi F., Giannakoulas G., Papadakis M. (2021). 2020 ESC Guidelines on sports cardiology and exercise in patients with cardiovascular disease. Eur. Heart J..

[32] McGregor G., Powell R., Kimani P., Underwood M. (2016). Exercise-based cardiac rehabilitation for coronary heart disease. Cochrane Database Syst. Rev..

[33] Niu S., Wang F., Yang S., Jin Z., Han X., Zou S., Guo D., Guo C. (2020). Predictive value of cardiopulmonary fitness parameters in the prognosis of patients with acute coronary syndrome after percutaneous coronary intervention. J. Int. Med. Res..

