# Supplementary material for: Prognostic Utility of a New Risk Stratification Protocol for Secondary Prevention in Patients Attending Cardiac Rehabilitation
Source: J Clin Med. 2022 Mar 30;11(7):1910. doi: 10.3390/jcm11071910 (PMC8999920; doi:10.3390/jcm11071910)
Supplement: Supplementary file 1 [file jcm-11-01910-s001.zip › jcm-1606629-supplementary.pdf]

Journal of
Clinical Medicine

Table S1. Baseline characteristics between patients who attended cardiac rehabilitation and the whole cohort of ambispective registry.

Characteristic/Variable	Total (*n* = 497)	Included (*n* = 238)	Not-Included (*n* = 259)	*p* Value
**Anthropometric**				
Age (years)	62.0 (54–72)	61.0 (53–69)	64.0 (55–75)	0.006
Women	93 (18.7)	36 (15.1)	57 (22.0)	0.049
BMI (Kg/m2)	27.1(24.8–30.1)	27.2(24.8–29.7)	27.0(24.6–30.4)	0.945
**Risk factors and comorbidities**				
Hypertension	281 (56.5)	120 (50.4)	161 (62.2)	0.008
Hyperlipidemia	324 (65.2)	157 (66.0)	167 (64.5)	0.728
Diabetes mellitus	143 (28.8)	49 (20.6)	94 (36.3)	<0.001
Current smoker	205 (41.2)	92 (38.7)	113 (43.6)	
Previous smoker > 1 year	146 (29.4)	77 (32.4)	69 (26.6)	0.529
Previous smoker < 1 year	20 (4.0)	10 (4.2)	10 (3.9)	
COPD	40 (8.0)	15 (6.3)	25 (9.7)	0.170
Cerebrovascular disease	23 (4.6)	10 (4.2)	13 (5.0)	0.665
Peripheral vascular disease	39 (7.8)	9 (3.8)	30 (11.6)	0.001
Anemia	87 (17.5)	26 (10.9)	61 (23.6)	<0.001
Chronic kidney disease	29 (5.8)	8 (3.4)	21 (8.1)	0.024
CV family history	92 (18.5)	45 (18.9)	47 (18.1)	0.827
Sudden death family history	11 (2.2)	8 (3.4)	3 (1.2)	0.095
**Diagnostics**				
STEMI	220 (44.3)	106 (44.5)	114 (44.0)	0.131
NSTEMI	174 (35.0)	91 (38.2)	83 (32.0)	0.131
Unstable Angina	103 (20.7)	41 (17.2)	62 (23.9)	
Previous ACS-MI	99 (19.9)	35 (14.7)	64 (24.7)	0.005
One vessel disease	263 (52.9)	135 (56.7)	128 (49.4)	
Two vessels disease	126 (25.4)	55 (23.1)	71 (27.4)	0.412
Three vessels disease	87 (17.5)	41 (17.2)	46 (17.8)	
Ejection fraction (%)	59 (52–62)	58 (52–63)	59 (53–62)	0.184
**Blood test**				
Glucose, mg/dL	108 (95- 135)	107 (95–131)	109 (95–135)	0.465
Glycated hemoglobin, %	5.7 (5.4–6.5)	5.6 (5.4–6.1)	5.9 (5.4–6.8)	0.001
LDL, mg/dL	109 (78–135)	113 (87–137)	102 (73–125)	0.006

Data are mean ± SD, median (IQR), or numbers (*n*) and percentages (%). ACS, Acute coronary syndrome-myocardial infarction; BMI, Body mass index; COPD, Chronic obstructive pulmonary disease; CV, Cardiovascular; MI, myocardial infarction; NSTEMI, non-ST-elevation myocardial infarction; STEMI, ST-elevation myocardial infarction.

Table S2. Baseline characteristics between patients who had cardiac readmission and those who did not.

Characteristic/Variable	Cardiac Readmission (*n* = 25)	Withouth Cardiac Readmission (*n* = 213)	*p* Value
**Anthropometric**			
Age (years)	64 (58–70)	61 (53–69)	0.101
Women	3 (12.0)	33 (16)	0.645
BMI (Kg/m2)	27.6 (24.5–29.6)	27.1 (24.8–29.6)	0.874
**Risk factors and comorbidities**			
Hypertension	15 (60)	105 (49)	0.311
Hyperlipidemia	11 (44)	146 (69)	0.014
Diabetes mellitus	4 (16)	45 (21)	0.549
Current smoker	4 (16)	88 (41.3)	
Previous smoker > 1 year	13 (52)	64 (30.0)	0.067
Previous smoker < 1 year	1 (4.0)	9 (4.2)	
COPD	3 (12.0)	12 (5.6)	0.215
Cerebrovascular disease	1 (4.0)	9 (4.2)	0.958
Peripheral vascular disease	0 (0.0)	9 (4.2)	0.295
Anemia	6 (24.0)	20 (9.4)	0.027
Chronic kidney disease	2 (8.0)	6 (2.8)	0.174
CV family history	2 (8.0)	43 (20.2)	0.141
Sudden death family history	0 (0.0)	8 (3.8)	0.324
**Diagnostics**			
STEMI	9 (36.0)	97 (45.5)	
NSTEMI	10 (40.0)	81 (38.0)	0.542
Unstable Angina	6 (24.0)	35 (16.4)	
Previous ACS-MI	6 (24.0)	29 (13.6)	0.165
One vessel disease	8 (32.0)	127 (59.6)	
Two vessels disease	8 (32.0)	47 (22.1)	0.033
Three vessels disease	9 (36.0)	32 (15.0)	
Ejection fraction (%)	57 (51–64)	58 (52–62)	0.268
**Blood test**			
Glucose, mg/dL	109 (102–156)	106 (95–128)	0.130
Glycated hemoglobin, %	5.7 (5.4–6.5)	5.6 (5.4–6.0)	0.414
LDL, mg/dL	97 (71–121)	113 (88–139)	0.063

Data are mean ± SD, median (IQR), or numbers (*n*) and percentages (%). ACS, Acute coronary syndrome-myocardial infarction; BMI, Body mass index; COPD, Chronic obstructive pulmonary disease; CV, Cardiovascular; MI, myocardial infarction; NSTEMI, non-ST-elevation myocardial infarction; STEMI, ST-elevation myocardial infarction.
